# Phosphorus Release and Regeneration Following Laboratory Lysis of Bacterial Cells

**DOI:** 10.3389/fmicb.2021.641700

**Published:** 2021-04-08

**Authors:** Aric H. Mine, Maureen L. Coleman, Albert S. Colman

**Affiliations:** ^1^Department of Earth and Environmental Sciences, California State University, Fresno, CA, United States; ^2^Department of the Geophysical Sciences, University of Chicago, Chicago, IL, United States; ^3^Department of Earth, Environmental and Planetary Sciences, Rice University, Houston, TX, United States

**Keywords:** phosphorus, lysis, regeneration, microbial loop, nutrient cycle

## Abstract

The availability of phosphorus limits primary production in large regions of the oceans, and marine microbes use a variety of strategies to overcome this limitation. One strategy is the production of alkaline phosphatase (APase), which allows hydrolysis of larger dissolved organic phosphorus (DOP) compounds in the periplasm or at the cell surface for transport of orthophosphate into the cell. Cell lysis, driven by grazing and viral infection, releases phosphorus-containing cell components, along with active enzymes that could persist after lysis. The importance of this continued enzymatic activity for orthophosphate regeneration is unknown. We used three model bacteria – *Escherichia coli* K-12 MG1655, *Synechococcus* sp. WH7803, and *Prochlorococcus* sp. MED4 – to assess the impact of continued APase activity after cell lysis, via lysozyme treatment, on orthophosphate regeneration. Direct release of orthophosphate scaled with cell size and was reduced under phosphate-starved conditions where APase activity continued for days after lysis. All lysate incubations showed post-lysis orthophosphate generation suggesting phosphatases other than APase maintain activity. Rates of DOP hydrolysis and orthophosphate remineralization varied post-lysis among strains. *Escherichia coli* K-12 MG1655 rates of remineralization were 0.6 and 1.2 amol cell^–1^hr^–1^ under deplete and replete conditions; *Synechococcus* WH7803 lysates ranged from 0.04 up to 0.3 amol cell^–1^hr^–1^ during phosphorus deplete and replete conditions, respectively, while in *Prochlorococcus* MED4 lysates, rates were stable at 0.001 amol cell^–1^hr^–1^ in both conditions. The range of rates of hydrolysis and regeneration underscores the taxonomic and biochemical variability in the process of nutrient regeneration and further highlights the complexity of quantitatively resolving the major fluxes within the microbial loop.

## Introduction

Phosphorus availability limits primary production in large regions of the oceans including major ocean gyres (Moore et al., 2013). Orthophosphate (P*i*), the main component of the analytically resolvable pool of P known as soluble reactive phosphorus (SRP), is the form of P most readily taken up by cells and incorporated into cellular biomass. SRP concentrations, as a surrogate for [P*i*], are often below 10 nM in surface waters of the Sargasso Sea during stratification and 9–40 nM in the North Pacific, while dissolved organic phosphorus (DOP) concentrations often reach 50 nM in the Sargasso and 150 nM in the North Pacific (Wu et al., 2000; Lomas et al., 2010; Karl and Björkman, 2015). Allochthonous supply of phosphorus to the ocean gyres comes from either upwelling or dust deposition, but the magnitude of these fluxes (21–31 mmol P m^–2^ yr^–1^) is a fraction of the demand inferred from measured productivity (135 mmol P m^–2^ yr^–1^) (McGillicuddy et al., 1998; Lomas et al., 2010). Hence, production of new biomass via autotrophic and heterotrophic activity of surface ocean communities is dependent on efficient recycling of phosphorus between cellular (particulate) fractions and dissolved pools.

The transformation of phosphorus from particulate to dissolved pools is facilitated through a number of processes: cellular exudation, grazing, autolysis, and viral lysis. The relative quantities and composition of dissolved organic matter (DOM) released by these mechanisms is poorly understood (Moran et al., 2016). The diversity of phosphorus-containing compounds released by these processes includes phospholipids, macromolecular nucleic acids, nucleotide sugars, and small molecule monomers (e.g., ATP). Upon release, this complex organic matter is further hydrolyzed by enzymes (e.g., phosphatases), either cell-associated (e.g., heterotrophs hydrolyzing various substrates for growth) or cell-free (e.g., stable proteins released by lysis) (Gobler et al., 1997; Middelboe and Lyck, 2002). Such hydrolysis reactions are critical for maximizing the availability of P to a wide range of microbial taxa, yet the rates and controls on these reactions following DOM release are poorly constrained.

Different taxa likely release different amounts and types of DOM given the diversity of metabolic strategies and cellular quotas observed in the surface ocean (Lomas et al., 2004; Martiny et al., 2013). Even within a given taxon, the physiological state of the cell influences the amount and stoichiometry of major nutrients and their allocation to distinct chemical pools. For example, cell quotas of phosphorus in *Prochlorococcus*, *Synechococcus*, and *E. coli* range from 0.5–2.0, 3.0–255.0, and 216–1000 amol P/cell, respectively, varying by orders of magnitude with growth conditions and strain (Fagerbakke et al., 1996; Bertilsson et al., 2003; Heldal et al., 2003). *Prochlorococcus* and *Synechococcus* use multiple strategies to reduce their cellular phosphorus demand under P-limited growth conditions, including the substitution of sulfolipids for phospholipids (Van Mooy et al., 2009). At the same time, *Synechococcus* and other phytoplankton and bacteria may increase intracellular stores of polyphosphate in P-limited environments (Orchard et al., 2010; Temperton et al., 2011; Martin et al., 2014). In addition to altering cell quotas for P, many organisms including picocyanobacteria upregulate transporters and enzymes such as alkaline phosphatase that enhance their ability to acquire P from both inorganic and organic sources (Donald et al., 1997; Moore et al., 2005; Ostrowski et al., 2010; Mazard et al., 2012; Krumhardt et al., 2013). Hence both microbial community structure and cell physiology are likely to influence the quantity and composition of P released by cell lysis, and its potential for downstream transformations.

Here, we sought to characterize the regeneration of phosphorus following microbial cell lysis using model bacterial strains. We quantified the release of phosphorus to organic and inorganic dissolved pools and monitored the kinetics of phosphorus transformation over time. Rates of regeneration are particularly important for oligotrophic aquatic regions in broadening our understanding of how nutrient-starved regions sustain primary production. In addition, rates of regeneration provide constraints for assessing the efficiency of the microbial loop as a driver for the export of carbon and ultimately modulation of climate.

## Materials and Methods

### Growth Conditions

Three model organisms were used in this study: *Escherichia coli* K-12 MG1655, *Synechococcus* sp. WH7803, and *Prochlorococcus* sp. MED4. Axenic cultures were grown in batch mode on specified minimal media and harvested during late log phase growth (Supplementary Table 1). Harvesting in this growth phase was necessary for sensitivity in the subsequent nutrient and enzymatic analysis. The limit of detection for phosphate analyses was 25 nm and 0.005 μmol/hr for APase activity. *E. coli* growth was monitored by OD_600_, *Synechococcus* WH7803 by phycoerythrin fluorescence (ex. 498nm, em. 573nm) and *Prochlorococcus* MED4 by chlorophyll *a* fluorescence (ex. 430nm, em. 664nm). *E. coli* was chosen due to its rapid growth and our ability to quickly test methodological approaches before extending them to more environmentally relevant organisms like *Prochlorococcus* and *Synechococcus.* All growth measurements were performed in a 96-well plate reader (Tecan Infinite 200 Pro).

Cyanobacterial cultures were grown in P-replete ASW and PRO 99 medium (Lindell et al., 1998; Moore et al., 2007) with orthophosphate as the sole phosphorus source (Supplementary Table 1). *E. coli* MG1655 was grown in M9 minimal medium with either orthophosphate (0.24 mM) or glycerophosphate (0.24 mM), as the sole phosphorus source. SRP-deplete conditions were achieved through *E. coli* growth in glycerophosphate-only M9 media to induce APase expression. To induce P-starvation in cyanobacterial cultures, cultures were grown in P-replete medium, harvested by centrifugation, and resuspended in medium with no added P. P starvation was assessed by assaying APase activity in whole cells, as described below. By this assay, P starvation was apparent in *Prochlorococcus* MED4 within 24–48 h, whereas *Synechococcus* WH7803 required up to 120 h, consistent with previous reports (Moore et al., 2005).

### Lysis Experiments

Phosphorus starvation was confirmed prior to lysis by assaying APase activity (see below). Just before lysis, cells were harvested by centrifugation at 8000 × *g* for 10 min and washed three times with 50mM Tris (pH 8) to remove residual phosphate from the growth medium. Triplicate cell culture aliquots (2ml; cell density > 10^9^cells/ml) were then lysed by incubation with egg-white lysozyme (Fisher) at a final concentration of 5mg/ml for 1 h at 37°C in 50mM Tris buffer (pH 8.0). Lysates were immediately assayed for APase activity and for soluble reactive phosphorus (SRP) and measured in triplicate. Samples for total dissolved phosphorus were frozen at −20°C until analysis. Cell counts were determined via flow cytometry with SYBR Gold staining. Effective lysis was confirmed by visual inspection using an epifluorescence microscopy. In addition, DNA extracted from cell lysates was quantified using a NanoDrop Spectrophotometer and compared against estimates of intracellular DNA/cell as a test of DNA yield and lysis efficiency > 90%.

### Lysate Incubations

Incubations were comprised of pelleted and rinsed cells lysed in a Tris buffer solution as mentioned above. Lysed cells and cellular debris were left suspended in a 2.0 ml microcentrifuge tube at 25°C for several days in the dark and monitored at 24–48 hr intervals. The suspension was centrifuged and the supernatant of this lysate solution was removed for nutrient and enzymatic assays. Some incubations were amended with DOP (final concentration 1mM; compounds detailed below) to compare rates of hydrolysis against unamended lysate incubations. Additional incubations were completed in solutions filtered through a 0.22 μm syringe filter to probe APase activity the particulate and dissolved (<0.2 lysate μm) fractions. A tabular summary of the different incubations, treatments, and the analyses performed is included (Supplementary Table 4).

### Enzyme and Nutrient Assays

APase activity was assayed using the fluorogenic substrate 6,8- Difluoro-4-methylumbelliferyl phosphate (DiFMUP) (Duhamel et al., 2010). Hydrolysis of DiFMUP produces the fluorescent product 6,8-Difluoro-7-hydroxy-4-methylcoumarin (DiFMU). DiFMU standards (0–4 μM) were prepared in sterile 50mM Tris pH = 8.0 and their fluorescence measured using a temperature controlled Tecan Infinite 200 Pro plate reader, with excitation at 358 nm and emission at 450 nm. Aliquots of intact cultures, 200 μl (to monitor P starvation during growth), or cell lysates (200 μl unfiltered lysate following lysozyme treatment) were incubated with 10 μM DiFMUP and fluorescence monitored over an assay period of 40 min. The rate of DiFMU production was calculated by the change in fluorescence over time. This concentration of substrate was determined to be saturating by varying DiFMUP concentration (0–100 μM) during enzyme assays and comparing subsequent rates. Thus, enzymatic activities presented here are interpreted as maximum activities.

Purified APase (Sigma) was used to test the enzyme activity and enzymatic lifetime when the enzyme is isolated from cell and cellular debris while being incubated in the presence of degrading proteins like Proteinase K. Purified calf alkaline phosphatase (Sigma Aldrich CAT: P6774) was assayed and contrasted against alkaline phosphatase activity from fresh *E. coli* lysate, with proteinase K added to both solutions. Proteinase K was used to test the ability to degrade alkaline phosphatase in lysate incubations given its broad specificity to degrade proteins. Alkaline phosphatase activity was measured via [SRP] as a proxy for hydrolysis via addition of glycerophosphate. The activation of proteinase K in solution was facilitated by increasing temperature to 55°C in a Tris solution, described above.

Soluble reactive phosphorus was measured spectrophotometrically using the phosphomolybdate blue method of Murphy and Riley (1962) adapted for this study by matrix matching of standards and samples. Potassium phosphate (KH_2_PO4) was used as an inorganic phosphate reagent, following 60°C oven drying for adsorbed water removal and Fisher egg-white lysozyme (CAT: 89833) was used as the lysing agent. Standard and sample solution matrices were the same compositionally, with same final concentration of lysozyme and analyzed in 50mM Tris, and diluted with DI water if necessary, to remain within the linear portion of the [SRP] (0–15 μM) standard curve. The dilution also reduced a spectrophotometric interference at 883nm related to the lysozyme.

Total dissolved phosphorus (TDP) was measured using published methods, where [TDP] = [SRP] + [DOP] (Monaghan and Ruttenberg, 1999). Samples were acid hydrolyzed and subsequently ashed, prior to final measurement as TDP. Standards (5’ Adenosine monophosphate, Na-Glycerophosphate, and sodium-pyrophosphate) and samples were treated with HCl (final concentration 0.1M HCl) and 50(w/v)% Mg(NO_3_)_2_ in combusted and acid cleaned glassware at 80°C for 24hrs and dried for at least 24hrs at 120°C. Following drying, samples were ashed in a muffle furnace at 500°C for 2hrs. Ashed material was dissolved in 3ml of 0.75M HCl and the pH adjusted to 2.0 with 1.0M HCl prior to final measurement as SRP, as described above. Hydrolysis and recovery experiments with the above-mentioned standards yield > 98% hydrolysis and TDP yield. Kimball glass scintillation vials capped with Teflon-lined caps proved most effective at limiting phosphorus contamination, while maintaining structural integrity, and were re-used following acid washing, DI rinsing, and liner replacement.

Model DOP compounds (1 mM) or pyrophosphate (0.5 mM) were added to pure-culture lysates to track the hydrolysis and breakdown of model compounds via SRP production. Concentration was determined to be saturating via the aforementioned procedure with DiFMUP. Hydrolysis rates of model DOP compounds were compared against measured rates of APase activity to explore whether additional phosphatases might be acting to hydrolyze DOP. Model DOP compounds included sodium glycerophosphate (CAT#: G9422; Fisher Scientific), sodium pyrophosphate decahydrate (CAT#: S390–500; Fisher Scientific), RNA isolated from yeast (CAT#: R5636; Sigma Aldrich), and yeast extracted 5’adenosine monophosphate (CAT: 01930; Sigma Aldrich). All compounds and chemicals were of ACS grade or higher.

*Z*-tests were completed on each time point of the lysate incubations. The mean of triplicate measurements was compared under the different phosphorus conditions to assess the statistical significance of the nutrient conditions through time. Z-scores were then used in the generation of a *p*-value as a test of the significance.

## Results

### Immediate Phosphorus Release During Cell Lysis Is Taxon-Specific and Physiology-Dependent

Immediately following lysis, P was quantified as total dissolved P (TDP), soluble reactive P (SRP), and dissolved organic P (DOP) (Table 1). The quantity of P released varied dramatically across taxa, with *E. coli* releasing three-fold more SRP per cell than *Synechococcus*, and 100-fold more than *Prochlorococcus*, when grown in P-replete conditions (Table 1). This SRP release scales with the total cellular quota of P, which ranges from nearly 1 fmol/cell for *E. coli* to about 200 amol/cell for *Synechococcus* and 32 amol/cell for *Prochlorococcus* in P-replete conditions (Supplementary Table 2). Similarly, the immediate SRP release in *E. coli* was 107 pmol/cell under p-replete conditions and 83 pmol/cell under p-deplete conditions.

**TABLE 1:** Initial phosphorus release upon cell lysis, in amol/cell.

**Strain**	**Condition**	**TDP**	**DOP (s.d.)**	**SRP (s.d.)**	**SRP ± s.d (% of TDP)**
*E. coli* K-12 MG1655	P-replete	n.d.	n.d.	105 (8.7)	n.d.
	P-deplete	n.d.	n.d.	83 (2.7)	n.d.
*Synechococcus* WH7803	P-replete	68 (6.2)	34 (6.3)	33 (0.5)*	49% (4.0)
	P-deplete	40 (7.5)	29 (7.5)	10 (0.6)	26% (4.1)
*Prochlorococcus* MED4	P-replete	0.81 (0.10)	0.06 (0.11)	0.86 (0.04)	107% (17)
	P-deplete	0.98 (0.10)	0.29 (0.12)	0.69 (0.06)	71% (5.2)

TDP, total dissolved phosphorus; SRP, soluble reactive phosphorus; DOP, dissolved organic phosphorus. DOP is calculated as TDP-SRP. n.d., not determined. Numbers in parentheses are one standard deviation of triplicate lysates. All replete and deplete conditions have *p*-values < 0.01, unless noted with an asterisk. * Indicates *p* < 0.1.

**FIGURE 1:**
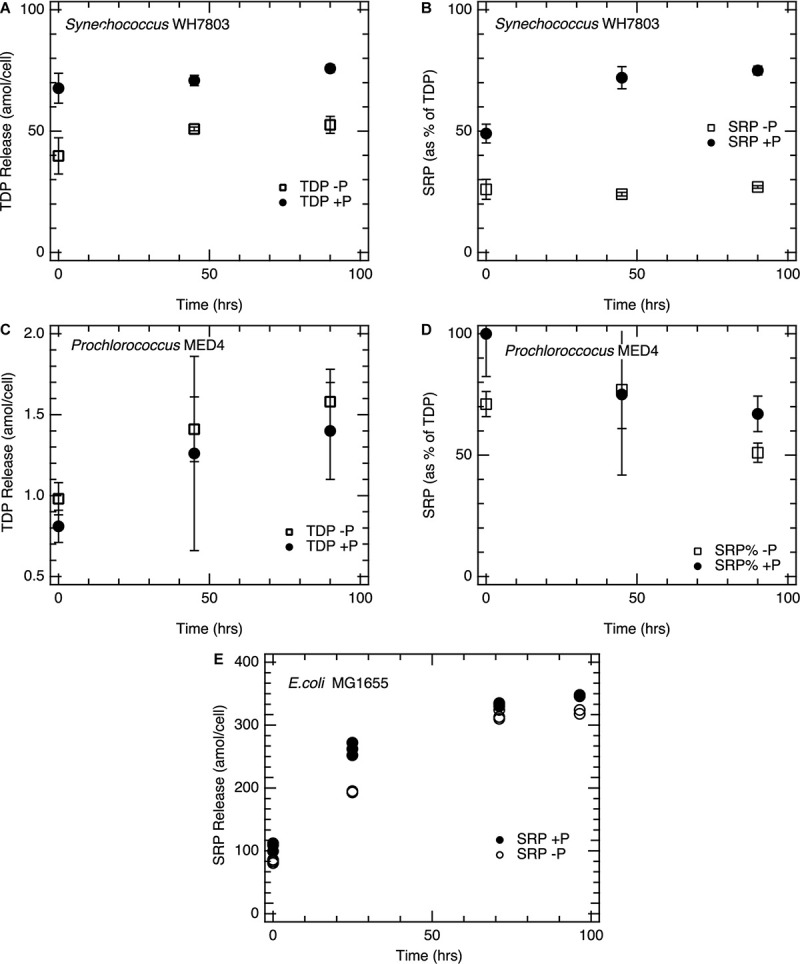
Total dissolved phosphorus and soluble reactive phosphorus release from cell cultures following lysis. **(A)** *Synechococcus* WH7803 TDP release; **(B)** *Synechococcus* WH7803 fraction of TDP as SRP; **(C)** *Prochlorococcus* MED4 TDP release; **(D)** *Prochlorococcus* MED4 fraction TDP as SRP. **(E)** SRP regeneration in *E. coli* incubations normalized to cell density. Open symbols represent lysates from P-deplete cells, filled symbols represent lysates from P-replete cells. All points represent the mean of triplicate measurements and error bars the standard deviation. *Synechococcus* WH7803 and *Prochlorococcus* MED 4 phosphorus replete and deplete conditions are statistically significant in the figures above; *p* < 0.01 and *p* < 0.1 after initial release, respectively.

Cellular P content is known to be reduced in P-limited picocyanobacteria (Bertilsson et al., 2003; Van Mooy et al., 2008). Consistent with this, P limitation caused a dramatic reduction in initial SRP release from *Synechococcus* WH7803 (Figure 1A and Table 1). The immediate release of SRP from P-replete cultures was 33 amol/cell compared to 10 amol/cell from P-deplete cultures. This reduction in SRP release largely accounted for the lower TDP release from P-deplete *Synechococcus* cells (40 amol/cell) compared to P-replete cells (68 amol/cell). SRP constituted a smaller fraction of TDP released from P-deplete cells (25%) compared to P-replete cells (49%).

*Prochlorococcus* MED4 cultures released far lower amounts of TDP upon lysis, consistent with their smaller cell size and lower cellular P quotas (Supplementary Table 2). We measured SRP release of 0.9 and 0.7 amol/cell for P-replete and P-deplete MED4 cultures, respectively (Table 1). This SRP release accounted for nearly all of the TDP released from MED4. TDP analysis on *Prochlorococcus* MED4 lysates appears more variable than other incubations, likely due to the precision of the assay at nanomolar P concentrations.

### Sustained Nutrient Regeneration During Lysate Incubations

Following lysis, regeneration of P continued for several days. TDP increased over time (Figures 1A,C), due to solubilization and enzymatic digestion of particulate cell debris. Likewise, SRP generally increased for several days as well (Figure 1). This accumulation of P as SRP was most pronounced in *E. coli*. For example, with the P-replete *E. coli* cultures, the initial post-lysis release of 105 amol SRP/cell increased to 350 amol/cell by day 5 (Figure 1E). Unlike the picocyanobacterial strains, *E. coli* exhibited similar SRP accumulation trajectories for both P-deplete and P-replete cells over the 96 hr period.

Picocyanobacteria showed lower per-cell increases in SRP and TDP over time, consistent with their lower cellular P quotas. In lysates from P-replete *Synechococcus*, DOP declined while SRP increased (Figure 1), consistent with a transformation from the former pool to the latter, such that by 96 h 75% of the TDP was SRP and only 25% was DOP. By contrast, DOP continued to make up over 70% of the TDP from P-deplete *Synechococcus*, suggesting that, unlike in the P-replete case, this DOP was not readily hydrolysable. Continued solubilization of particulate P released additional TDP over the 4 day incubation.

*Prochlorococcus* release of SRP and TDP was not as statistically distinct when comparing P-replete and P-deplete conditions (*p* < 0.1). *Prochlorococcus* incubations exhibited a more irregular pattern of SRP accumulation over time (Figures 1C,D and Table 1). In lysates from P-replete *Prochlorococcus*, there was a significant increase in TDP (transformed from the particulate phase) over time, mostly in the form of DOP; this DOP was not readily hydrolzyed to SRP, hence the fraction of SRP declined over time (Figures 1C,D). The fraction of cellular P regenerated in dissolved form during the 4 day incubation reached about 15% for P-deplete and 3–5% for P-replete *Prochlorococcus* cultures.

### Alkaline Phosphatase Activity Following Lysis

**FIGURE 2:**
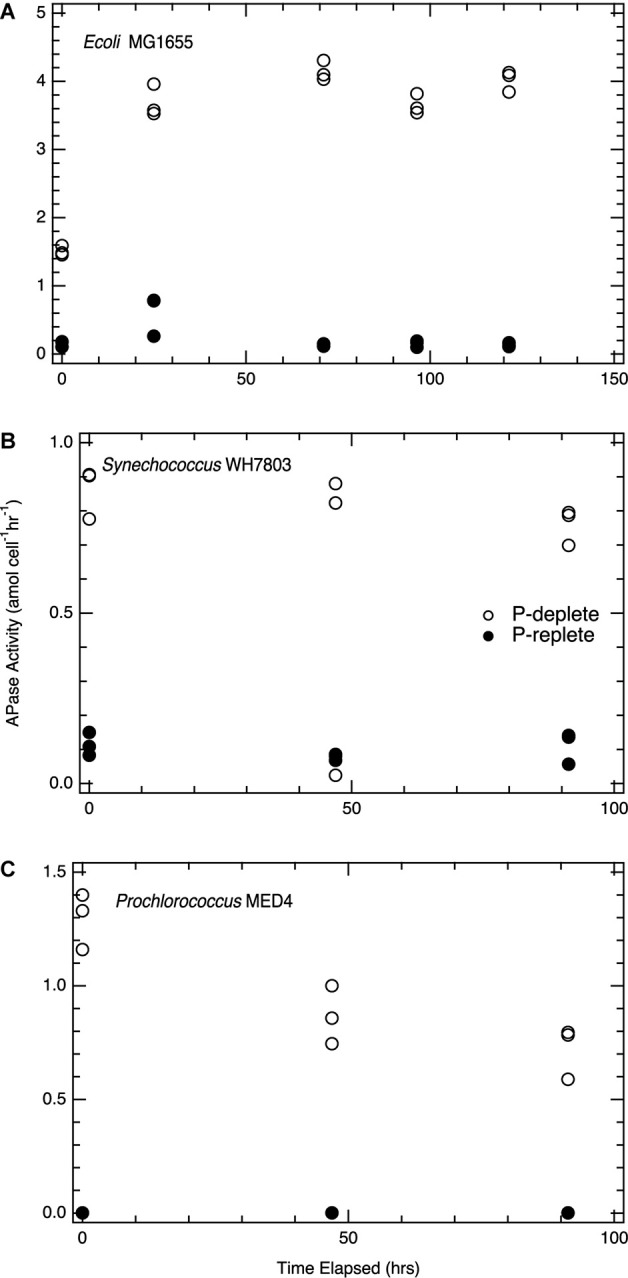
Alkaline phosphatase activity following cell lysis. **(A)** *E. coli*; **(B)** *Synechococcus* WH7803; **(C)** *Prochlorococcus* MED4. Filled circles, cells grown in P-replete conditions; open circles, cells grown in P-deplete conditions. DiFMUP concentrations for incubations were 10 μM and all enzymatic rates are determined as maximum activities, where substrate is not deplete. For each time point, triplicate lysate incubations were measured, and the analytical error bars for each measurement are within the size of the symbols. Replete and deplete phosphorus conditions are statistically significant for the all the strains above; *p* < 0.01.

Enzymatic activity following lysis was sustained over the 96hr period incubation with rates of activity linked to nutrient status. In all three taxa, APase activity, measured via DiFMUP hydrolysis, was significantly higher (*p* < 0.01) in lysates from P-deplete cells compared to P-replete cells (Figure 2), consistent with the known upregulation of APase under P-limited conditions (Torriani-Gorini et al., 1994; Donald et al., 1997; Moore et al., 2005). This activity persisted for at least 96 h following cell lysis (Figure 2). The continued activity of APase provides an important mechanism to transform the dissolved and particulate nutrient pools.

Alkaline phosphatase activity in lysates, as measured with the substrate DiFMUP, was distinct from rates of DOP hydrolysis and SRP production in these same lysates. Although APase activity was low and near the limit of detection in lysates from P-replete cells of all three taxa (Figure 2), SRP continued to be regenerated (Figure 1). *Synechococcus* lysates showed six-fold higher APase activity from P-deplete cells than from P-replete cells. Yet SRP did not accumulate significantly over time in lysate incubations of P-deplete cultures; most TDP remained as DOP (Figures 1B, 2B). In contrast, nearly as much SRP was regenerated post-lysis in the P-replete *Synechococcus* lysate, via hydrolysis of DOP, as had been released during the initial lysis step, despite very low APase activity. This finding of SRP regeneration with limited APase activity in the lysate is further explored below.

### Enzymatic Activity Investigated Through Filtered vs. Unfiltered Lysate Incubations and Proteinase *K*-Tests

We posited freshly lysed cells and recently generated debris might house APase activity given the membrane-bound nature of the enzyme. Consistent with this prediction, APase activity in the dissolved (<0.22 μm) lysate fraction was 10-fold lower than unfiltered *E. coli* lysate (1.2 ± 0.11 μmol L^–1^ hr^–1^ vs. 12 ± 0.17 μmol L^–1^ hr^–1^).

**FIGURE 3:**
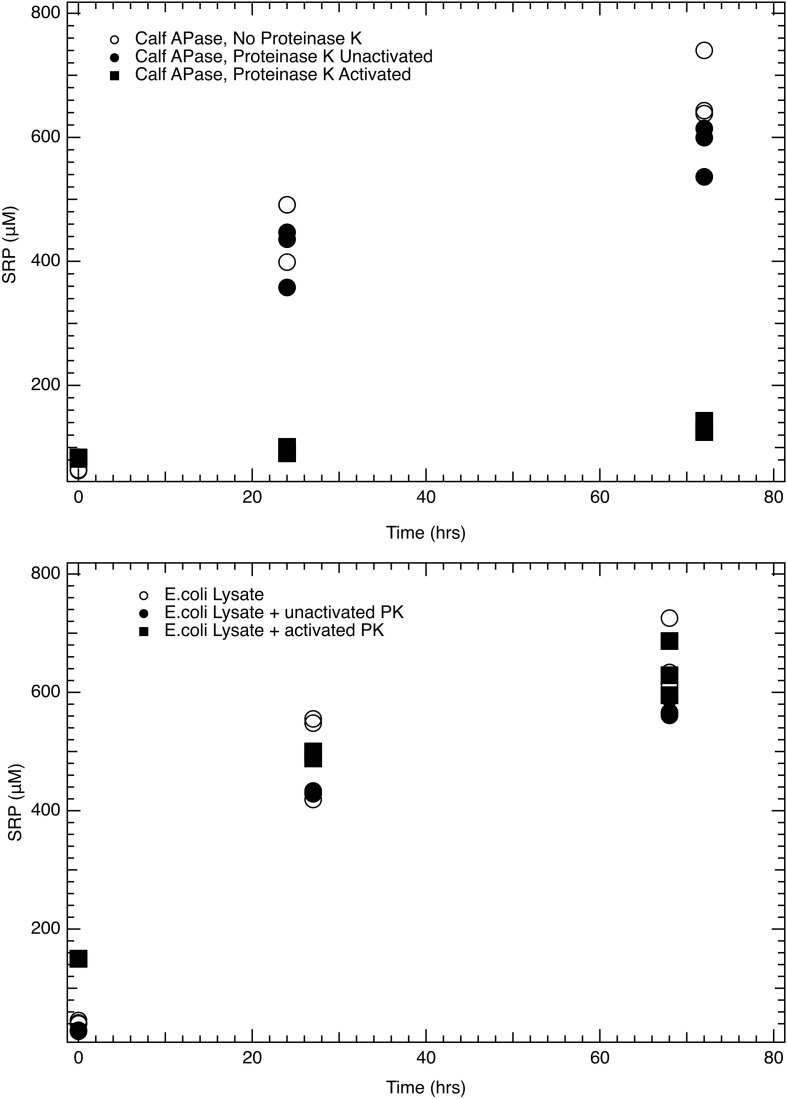
Proteinase K treatment of purified enzyme and lysate: **(Top)** Change in soluble reactive phosphorus (SRP) concentration during incubation of a 1 mM glycerophosphate solution with calf purified APase under three conditions: (1) open circles represent APase enzyme with no proteinase K present; (2) filled circles represent APase with Proteinase K present, but no temperature activation of the enzyme; (3) filled squares represent incubations where APase is incubated with Proteinase K activated at 55°C. **(Bottom)** Change in SRP concentration during similar incubations in which calf APase is replaced by *E. coli* lysate in the incubations, again spiked to 1 mM glycerophosphate. Open circles represent incubations with only *E. coli* lysate, filled circles represent *E. coli* lysate incubated with unactivated proteinase K, and filled squares represent incubations with *E. coli* lysate and Proteinase K activated at 55°C. Purified APase showed a two order of magnitude reduction in activity when proteinase K was activated at 55°C. When incubated at room temperature, unactivated proteinase K had no observable effect on diminishing APase activity (top). Proteinase K had no effect on APase activity in fresh *E. coli* lysate even when it was heat activated at 55°C (bottom). Analytical errror bars are within the size of the symbol. Differences between unactivated and activated Proteinase K treatments are statistically significant *P* < 0.01. There is no statistically significant difference between the *E. coli* lysate conditions.

Lysate and purified enzyme showed contrasting results for the fate of enzyme activity when incubated with Proteinase K (Figure 3). Purified alkaline phosphatase activity was diminished immediately following temperature activation of proteinase K at 55°C, while APase activity in *E. coli* lysate continued uninhibited by proteinase K activation. However, Proteinase K in solution prevented the direct measurement of APase activity via a fluorophore. *E. coli* lystate unaffected by Proteinase K addition furthers supports our suggestion of the membrane localization of the enzyme.

### Hydrolysis of DOP Model Compounds

**FIGURE 4:**
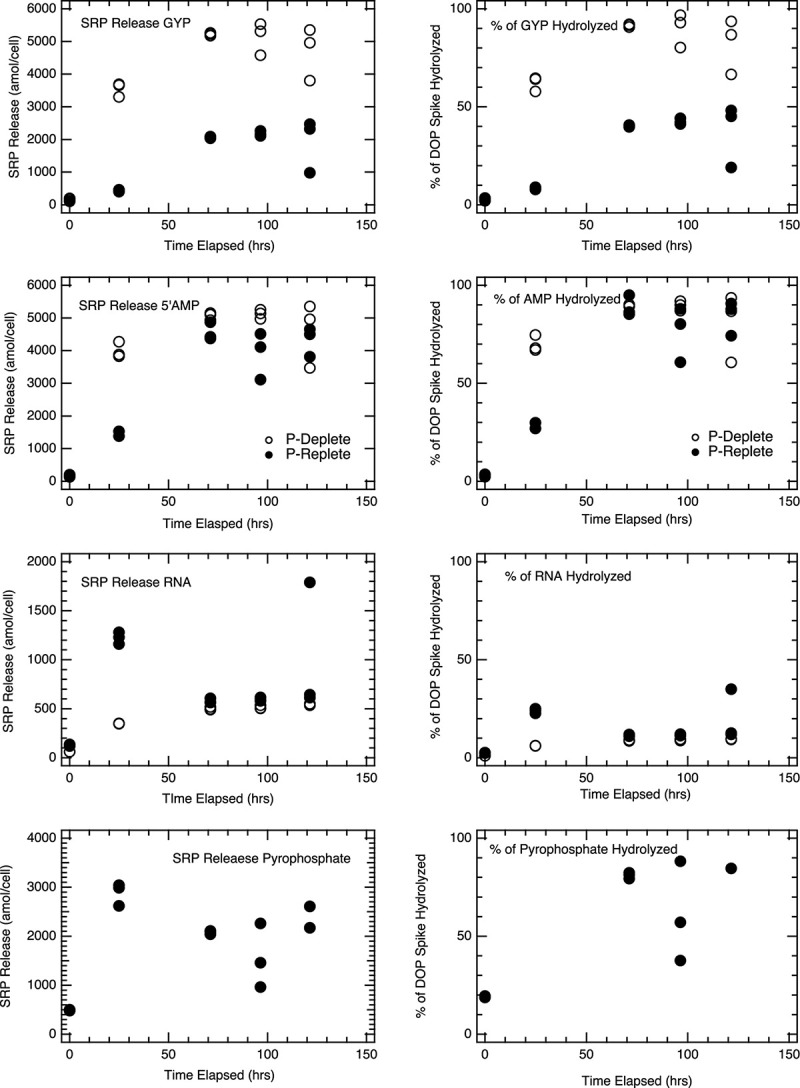
Hydrolysis of model DOP compounds in *E. coli* lysates: *E. coli* MG1655 P-Deplete and P-Replete lysates incubated with a range of DOP compounds (1 mM DOP concentration in lysate solution at beginning of incubation) were monitored for [SRP] over time. Model DOP compounds used include glycerophosphate (GYP), 5’adenosine monophosphate (5’AMP), ribonucleic acid (RNA), and pyrophosphate (Pyro-P). For each time point, triplicate lysate incubations were measured, and the analytical error bars for each measurement are within the size of the symbols. SRP release in GYP incubations is the only treatment above where p-conditions represent statistical differences in SRP regenerated; *p* < 0.01.

Model DOP compounds were used as analogs to freshly lysed organic matter. We spiked *E. coli* cell lysates with model DOP compounds and monitored SRP accumulation over time. Lysates from both P-replete and P-deplete *E. coli* exhibited the ability to hydrolyze the range of DOP compounds we tested (Figure 4). However, the fraction of the DOP spike hydrolyzed varied across compounds and with cell physiology. Addition of 5’-AMP, for example, led to nearly complete phosphorus release as SRP in lysates from both P-replete and P-deplete cells after 96 h, while only 10–15% of P added as RNA was fully hydrolyzed to SRP (Figure 4). Glycerophosphate (GYP) hydrolysis (measured by an increase in SRP) was nearly 100% in lysates from P-deplete cells but only 50% in lysates from P-replete *E. coli* cells (Figure 4, top panels); despite the fact that the resultant 1 mM [SRP] was enough to inhibit significantly APase activity (experiments not shown).

The efficiency of SRP regeneration from P-deplete *E. coli* cells was higher than from P-replete cells. This is consistent with the 10 to 40-fold higher APase activity in P-deplete cultures (Figure 4). In these P-deplete lysate incubations, the rates of GYP hydrolysis were over two orders of magnitude higher than those of DiFMUP for the APase assay. In the P-replete lysate incubations, GYP hydrolysis rates were still high even though APase activity was minor. 5’-AMP incubations similarly showed much more rapid hydrolysis in P-deplete culture lysates, though the hydrolysis of the spike essentially went to completion in both P-deplete and P-replete lysates within 2–3 days. Extensive hydrolysis of GYP (P-deplete) and 5’-AMP (both P-deplete and P-replete) produced APase inhibiting concentrations of SRP. RNA associated P was surprisingly stable, with only about 10% of the spike fully hydrolyzed to liberate phosphate. It is possible that the ribonucleases and auto-hydrolysis of RNA were inhibited in the lysate solution or that polynucleotides were partially stabilized in the lysate solution. Pyrophosphate stability was only tested in P-replete lysate. It rapidly hydrolyzed (<24 hrs) to yield essentially all of its P as SRP.

**FIGURE 5:**
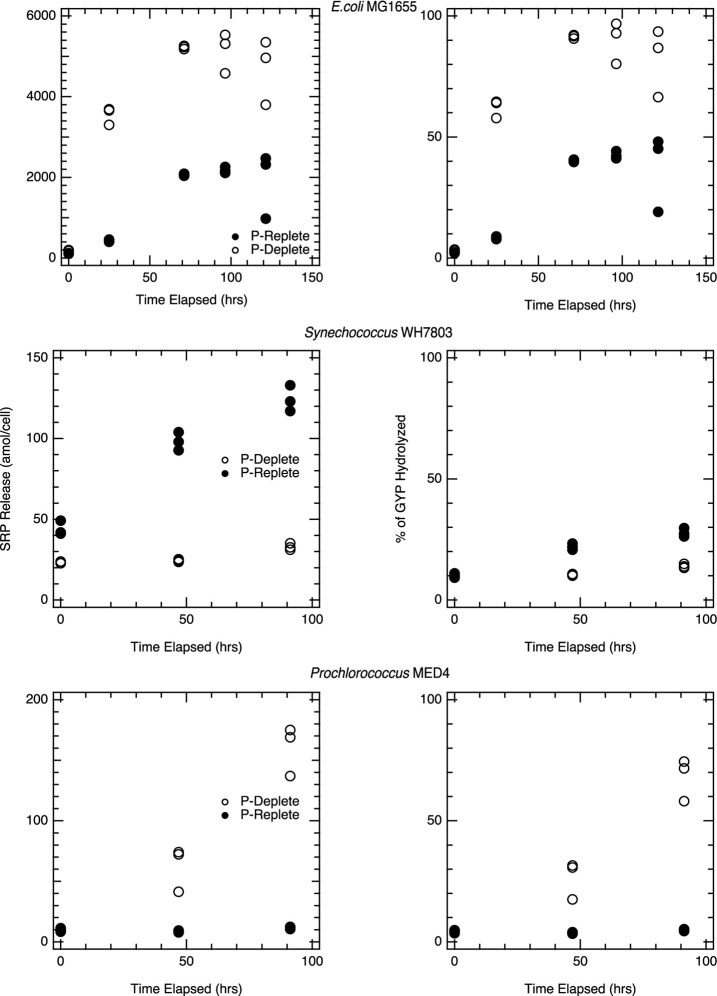
Hydrolysis of GYP during incubations with pure culture lysate: Figures represent pure culture lysates, *E. coli* K-12 MG1655 (top), *Synechococcus* WH7803 (middle) and *Prochlorococcus* MED4 (bottom) in which the lysate was spiked to a 1mM initial glycerophosphate concentration and was monitored for at least 96hrs. For each time point, triplicate lysate incubations were measured, and the analytical error bars for each measurement are within the size of the symbols. Each phosphorus treatment is statistically significant for the strains above; *p* < 0.01.

Similar experiments with picocyanobacterial cell lysates were completed in the presence of GYP. Lysates from P-deplete *Prochlorococcus* MED4 cells showed a rapid increase in SRP after GYP addition, with a spike of 1mM GYP resulting in over 700 μM SRP after 96 h (Figure 5). Lysates from P-replete *Prochlorococcus*, on the other hand, showed no corresponding increase in SRP, consistent with their much lower APase activity (Figure 5). APase assays and GYP-spike incubations produced results consistent with one another. Initial APase activity was higher per cell in the P-deplete *Prochlorococcus* lysate than for *Synechococcus* and P-replete *E. coli* (Figure 2), whereas P-replete *Prochlorococcus* had undetectable APase activity. The P-deplete *Prochlorococcus* APase activity declined steadily over the 4-day incubations, from an initial rate of roughly 1.3 amol cell^−1^ hr^−1^ to 0.7 amol cell^−1^ hr^−1^ by the end. Such declines were not observed in *Synechococcus* nor *E. coli*. GYP hydrolysis rates were rapid and appeared to accelerate over the course of the incubations of P-deplete *Prochlorococcus* lysate. Whereas GYP hydrolysis was negligible in lysate from P-replete cultures. Hydrolysis rates of GYP (Figure 5) were within a factor of two of the APase activities (Figure 2) for *Prochlorococcus*, while *E. coli* and *Synechococcus.* showed hydrolysis rates more distinct from GYP rates, five to ten-fold greater than APase activities.

Alkaline phosphatase assays and DOP-spike incubations on the *Synechococcus* lysate yield more complex results. *Synechococcus* lysates from P-replete cells showed greater SRP accumulation than lysates from P-deplete cells in the presence of GYP (Figure 5), despite higher APase activity in P-deplete cells. APase activity was eight times higher in P-deplete lysate than P-replete (Figure 2). Yet hydrolysis of the naturally produced DOP that was released and incubated in the unspiked lysate (Figure 1) and hydrolysis of GYP in the GYP-spiked lysate (Figure 5) were much more rapid in the P-replete lysate than P-deplete. *Synechococcus* APase is expected to hydrolyze GYP, so the roughly order of magnitude slower rate of GYP hydrolysis than DiFMUP hydrolysis in the P-deplete lysate conflicts with our expectation. We surmise that there are other enzymes that play a stronger role in hydrolyzing GYP than APase. This is also indicated by the significant hydrolysis of GYP by P-replete *E. coli* lysate (Figure 5) in which assayed APase activity was low (Figure 2).

## Discussion

### Laboratory Lysis and Nutrient Regeneration

Cellular lysis is an important route for nutrient regeneration in aquatic microbial ecosystems, especially in oligotrophic regions that are heavily reliant on internal cycling of nutrients to sustain productivity. Our cell lysis and lysate incubation experiments show that both direct release of cytosolic SRP and the sustained catalytic activity of phosphohydrolase enzymes, lead to the hydrolysis of DOP compounds to liberate SRP and are important for returning nutrients to bioavailable forms. This was true for cultures of *E. coli*, *Synechococcus* WH7803, and *Prochlorococcus* MED4, though the patterns of P release were different among strains and were sensitive to the nutrient status of the cells prior to lysis.

Soluble reactive phosphorus release from *E. coli*, *Synechococcus* WH7803, and *Prochlorococcus* MED4 following lysis included a rapid burst of presumably cytosolic SRP. There could also be a contribution of SRP from DOP compounds that were very unstable and hydrolyzed on the time scale of the lysis reaction and time zero sampling (roughly an hour-long process). We do not consider this potential flux separately from the pulsed release of cytosolic SRP in the following discussion. Although initial SRP release was greater from P-replete cells, the efficiency of P-release was greater from the lower P-content, P-deplete cells. In both P-deplete and P-replete cultures, the initial burst of SRP release was generally followed by significant sustained release of SRP for 2–4 days (Figure 3), with most of the release happening in the first 2 days.

Published nutrient quota measurements provide useful context to contrast per cell P inventory for strains and regeneration trajectories determined above. Cyanobacterial strains have documented flexibility in cellular stoichiometry (Bertilsson et al., 2003; Krumhardt et al., 2013; Martin et al., 2014), in part owing to the ability of some strains to produce polyphosphates.

Polyphosphate storage is well documented in *E. coli* and *Synechococcus* strains and in other surface ocean microbial communities (Rao et al., 1998; Diaz and Ingall, 2010; Martin et al., 2018). Although *Prochlorococcus* genomes contain genes related to polyphosphate breakdown and/or storage (e.g., *ppk*, *ppx)* polyphosphate storage is not as well documented in this cyanobacterium (Heldal et al., 2003; Martiny et al., 2006; Temperton et al., 2011). Previous laboratory *Synechococcus* WH7803 cultures found P quota to be strain specific with polyphosphate storage resulting in at least 2-fold increases in cellular P-quota (Heldal et al., 2003). Our immediate SRP release from P-replete and P-deplete *Synechococcus* cultures constitutes 17% and 5% of cellular P, respectively, when compared against documented polyphosphate storing laboratory *Synechococcus* WH7803 (Scanlan et al., 1993; Supplementary Table 2). The immediate SRP release as a fraction of cellular P increases to 33% and 15% of *Synechococcus* WH7803 cultures when comparing against P inventories excluding polyphosphates. Normalizations of phosphorus release to other cellular characters, like biovolume, were considered, however, data are limited on field populations (Supplementary Table 3). Compared to lower-limit published P cell quotas, our results indicate roughly 40% of the cellular P in *Synechococcus* P-deplete cells may be immediately released upon lysis into the TDP pool, compared to approximately 26% of the cellular P in P-replete cells (Supplementary Table 2). Similarly, *Prochlorococcus* releases > 70% of P as DOP immediately following lysis. Relative to published cellular P quotas for MED4, at most 10% of cellular P was released immediately by lysis into the TDP pool (Bertilsson et al., 2003; Krumhardt et al., 2013; Martiny et al., 2016; Supplementary Table 2). These findings support our hypothesis that DOP is a major phosphorus reservoir that ultimately supplies a significant regenerative flux post-lysis, while the initial pulse of SRP is combination of intracellular P and solubilized intracellular components (i.e., polyphosphate).

Alkaline phosphatase was our enzyme of focus due to its putative link to nutrient stress status, its broad specificity for phosphorus-rich substrates, and our ability to quantify activity with a straightforward fluorophore. APase activity persisted for all three strains over the 4 day duration of the lysate incubations with only slight declines, largely unchanged from the initial activity levels on harvested live cells. Glycerophosphate (GYP), 5’ AMP, and pyrophosphate were readily hydrolyzed even in lysate from P-replete cultures for which APase activity was minimal. RNA hydrolysis was relatively inefficient compared to the other compounds tested. Moreover, APase activity differences in Proteinase K incubations supports our hypothesis that 1) APase in the membrane-bound fraction of the unfiltered lysate was shielded from proteinase K degradation, analogous to debris-bound enzymes in natural aquatic environments; and/or 2) other membrane-specific phosphohydrolases are equally capable of DOP hydrolysis, but shielded from proteinase K as well. The overall set of observations indicates that phosphohydrolase enzymes in addition to APase remained active in the lysates. Most APase activity was linked to cellular particulate debris, and APase activity remained associated almost entirely with cell membranes.

Discrepancy in the GYP-derived rates of hydrolysis and those from DiFMUP suggest that additional phosphatases that do not hydrolyze DiFMUP could be important for releasing SRP in lysates from P-replete cells. In field studies, this implies that APase assays might underestimate phosphohydrolytic capacity and response to nutrient stress. Therefore, other phosphatases remain important in the hydrolysis of DOP compounds with a range of specificities and affinities for substrate (Button et al., 2004). By extension, the enzymes responsible for DiFMUP hydrolysis do not necessarily act proportionately on all DOP compounds present in cell lysates. Clearly, taxa, the compounds strains generate, and their physiological state have an impact on the nutrient release and the ultimate path of nutrient regeneration.

### Relating Experimental Results to Open Ocean Nutrient Cycling

#### Direct Release of SRP

Previously measured cellular P inventories vary 40 and 15-fold in surface ocean samples in which *Synechococcus* and/or *Prochlorococcus*, respectively, are the most abundant cyanobacteria. In laboratory cultures, these cellular P inventories vary by strain 3–5 fold in *Synechococcus* WH7803, WH8103, and WH8012, and in *Prochlorococcus* MED4 [summarized in Supplementary Table 3; data from Cuhel and Waterbury (1984); Vrede et al. (2002); Bertilsson et al. (2003); Heldal et al. (2003); Grob et al. (2013); Krumhardt et al. (2013); Baer et al. (2017)]. The typical P content of cultured *Synechococcus* WH7803 is 100–250 amol/cell scaling with nutrient status (Cuhel and Waterbury, 1984; Heldal et al., 2003) which we use in our discussion in the absence of direct measurements of the cellular P content of our intact cultures. These inventories are higher than those for many other cultured *Synechococcus* strains (e.g., WH8102; Bertilsson et al., 2003; Heldal et al., 2003).

Direct SRP release during lysis appears to be a modest but important regeneration flux for P in marine ecosystems. In this study, the immediate dissolved P release as SRP from P-replete *Synechococcus* WH7803 was 33 amol/cell for P-replete cultures and 10 amol/cell for P-deplete cultures. The initial SRP release therefore spans a relatively narrow range of ca. 10–15% of the P needed as a constituent of a new P-replete cell of comparable composition. However, if over the course of a bloom nutrient concentrations decline, then our results suggest lysis of a P-replete cell could immediately provide a third of the P as SRP required for the biomass of a new P-stressed cell.

#### Sustained Hydrolysis of DOP Following Lysis

Alkaline phosphatase activity measurements in marine microbiology have generally been used as a qualitative or semi-quantitative indicator of nutrient stress and enzymatic potential. The per cell APase activities that we measured (generally 0.6 – 1.4 amol cell^−1^ hr^−1^ for P-stressed *Synechococcus* and *Prochlorococcus*) are similar to those observed in natural surface ocean samples. Casey et al., 2010 measured APase activity to be 0.008 – 0.11 amol cell^−1^ hr^−1^ in the Sargasso Sea. Baltar et al., 2009 measured APase activity to be 1.3 – 5.2 amol cell^−1^ hr^−1^ for a deeper microbial community (200–1000m water depth in the subtropical Atlantic). Picocyanobacteria are dominant members of the microbial community throughout the nutrient-deplete surface ocean; mean euphotic zone *Prochlorococcus* cell densities in the NPSG are 2 × 10^5^ cells/ml and 1 × 10^4^ cells/ml in the Sargasso Sea (Björkman et al., 2000; Martiny et al., 2016). Mean *Synechococcus* abundances are generally lower in the NPSG and Sargasso Sea, ∼10^3^ cells/ml (Björkman et al., 2000; Martiny et al., 2016). The highest rates above are similar to those estimated for P*i* community uptake in the North Pacific Subtropical Gyre (St. ALOHA: 3 – 8.2 nM/day, Björkman et al., 2000; 0.8–9.1 nM/day, Björkman et al., 2012; 1 – 15 nM/day (Björkman et al., 2018), and DOP production rates (0.3 – 0.82 nM/day (Björkman et al., 2000). They are also similar to the lower end of the range of P*i* community uptake (3.12–50.4 nM/day) reported by Lomas et al., 2014 for a transect across the western North Atlantic Subtropical Gyre.

It is not straightforward to translate these rates into actual hydrolysis rates of naturally occurring DOP compounds. We are not aware of other attempts to assess the potential for regeneration of SRP from incubation experiments of dead or lysed cells with individual DOP compounds nor with natural mixtures of DOP produced from the lysis of the culture itself.

An alternative way to consider the importance of DOP hydrolysis by enzymes released during cell lysis is that the incubated cellular debris in our experiments retained its phosphohydrolase activity for the full 4 day duration of our experiments (Figures 1–5). In some cases, the enzyme activity increased markedly following lysis (e.g., P-deplete *E. coli* APase assay, Figure 2). In other cases, enzyme activity declined modestly, though never more than by a factor of 2 (e.g., P-deplete *Prochlorococcus* APase assay, Figure 2). If we consider the phosphohydrolytic activity to be retained mainly in membrane-fragments, then the importance of these dead cells is in direct proportion to their abundance relative to live cells. Previous reports have suggested that “ghost” cells (non-nucleoid cells) can make up a substantial portion of bacterioplankton-sized and shaped particles in certain environments (Supplementary Figure 2; Zweifel and Hagstrom, 1995; Del Giorgio and Gasol, 2008). Our results bolster the case for the catalytic potential and biogeochemical importance of both intact and degraded cellular debris (Mason et al., 1986; McCarthy et al., 1998). The main removal mechanisms for this debris are likely protistan grazing and disintegration and solubilization into colloidal or dissolved organic fractions. The importance of this cellular debris is likely greater during post-bloom and steady-state conditions than during blooms, though the sustained debris-associated regeneration of nutrients during these intervals may contribute to future blooms.

Viruses are a principal mechanism for phosphorus release aquatic environments and the diminishment of blooms. Model estimates of nutrient release following viral lysis estimate dissolved nitrogen and phosphorus release ranging from 0.2–1.2 μmol N L^–1^ hr^–1^ to 0.04–0.23 μmol P L^–1^ hr^–1^ (Wilhelm and Suttle, 1999) while measured rates of nutrient regeneration of ammonium following viral lysis are 0.024 μmol N L^–1^ hr^–1^ (Shelford et al., 2012). These bulk regeneration rates are in the same range as our strain-specific rates presented here. However, even before lysis, viral infection induces shifts in cellular metabolism, such as upregulation of nutrient-specific host genes (Thompson et al., 2011; Zeng and Chisholm, 2012; Puxty et al., 2016; Monier et al., 2017; Fang et al., 2019; Howard-Varona et al., 2020). The ability of phage to manipulate host metabolism has significant implications for nutrient cycles (Zimmerman et al., 2020). Viral lysis remains an important mode of lysis requiring further quantification of nutrient release and regeneration fluxes.

The balance between direct release of cytosolic phosphate and extracellular hydrolysis of DOP to yield phosphate plays a major role in governing the stable isotope ratio of phosphate oxygen (Colman et al., 2005; Paytan and McLaughlin, 2007). Intracellular phosphate is believed to be in isotopic equilibrium with ambient water (Blake et al., 2005; Chang and Blake, 2015), whereas extracellular enzymatic hydrolysis of DOP compounds results in substantial kinetic isotope effects and disequilibrated phosphate (Blake et al., 2005; Liang and Blake, 2006). These lysis incubations reveal that in the open ocean, likely < 50%, and in P-stressed picocyanobacterial communities, perhaps < 5% of cellular P inventories are released directly as cytosolic SRP during cell lysis. The potential exists for cellular debris to catalyze the production of substantially more SRP from extracellular hydrolysis, which would shift dissolved phosphate away from isotopic equilibrium with ambient seawater (Colman and Mine, 2016; Supplementary Figure 1).

## Conclusion

Cellular lysis is an important route for nutrient regeneration in aquatic microbial ecosystems, especially in oligotrophic regions that are heavily reliant on internal cycling of nutrients to sustain productivity. Our cell lysis and lysate incubation experiments show that both direct release of cytosolic SRP and the sustained catalytic activity of phosphohydrolase enzymes, coupled to the hydrolysis of DOP compounds to liberate SRP, are important for returning nutrients to bioavailable forms. Patterns of P release and regeneration were taxon-specific and nutrient status dependent. Our overall set of observations indicates that phosphohydrolase enzymes in addition to APase remained active in the lysates. The sustained catalytic activity of phosphohydrolase enzymes after cell death and lysis, their frequent association with cell membranes, and the resultant sustained production of SRP in incubated lysate solutions indicate that cellular debris is a potent mediator of biogeochemical fluxes. Discrepancies in enzymatic rates determined with fluorophores versus model DOP compounds suggests field-determined rates of enzymatic activity are more variable, and instead a function of enzymatic affinity for substrate. Preliminary estimations suggest SRP regeneration fluxes attendant to lysis and sustained phosphohydrolytic activity are of the same magnitude as microbial community DOP production rates and SRP uptake rates observed in oligotrophic subtropical gyres. Phosphohydrolytic enzyme activity endured after lysis aligns with observed decoupling between P and C throughout the ocean water column. Orthophosphate is liberated to support additional productivity and carbon export leaving high C:P dissolved organic matter behind.

## Data Availability Statement

The original contributions presented in the study are included in the article/Supplementary Material, further inquiries can be directed to the corresponding author/s.

## Author Contributions

Each author contributed to the design of the experiments in this article. Data collection was completed by AM under the supervision of MC and AC. The synthesis of the data, and the completion of the manuscript was a collaborative projected with contributions from all authors.

## Conflict of Interest

The authors declare that the research was conducted in the absence of any commercial or financial relationships that could be construed as a potential conflict of interest.

## Supplementary Material

Supplementary Figure 1 Conceptual Diagram of Cellular Phosphorus Cycling: Our study focuses on quantifying and mechanistically understanding phosphorus release and regeneration fluxes within the microbial loop. The major fluxes at the cellular level for phosphorus cycling are shown above alongside the pathways and reactions that transform phosphorus between phosphorus pools.

Supplementary Table 1 Media Composition (Lindell et al., 1998; Moore et al., 2007).

Supplementary Table 2 Compilation of Cultured Cellular P Quotas: Collection of published cellular p contents (Qp) for Prochlorococcus and Synechococcus strain

Supplementary Table 3 Compilation of Cultured Cyanobacterial Biovolumes: Collection of published cellular biovolume for surface ocean populations of *Prochlorococcus* and *Synechococcus*. Not reported – (n.r).

Supplementary Table 4 Summary of Experiments: Below is a tabular presentation of the experiments performed, alongside the analyses and treatments for each strain studied.

## References

[B1] Baer S. E. Lomas M. W. Terpis K. X. Mouginot C. Martiny A. C. (2017). Stoichiometry of *Prochlorococcus*, *Synechococcus*, and small eukaryotic populations in the western North Atlantic Ocean. *Environ. Microbiol.* 19 1568–1583. 10.1111/1462-2920.13672 28139885

[B2] Baltar F. Arístegui J. Sintes E. Van Aken H. M. Gasol J. M. Herndl G. J. (2009). Prokaryotic extracellular enzymatic activity in relation to biomass production and respiration in the meso- and bathypelagic waters of the (sub)tropical Atlantic. *Environ. Microbiol.* 11 1998–2014. 10.1111/j.1462-2920.2009.01922.x 19508555

[B3] Bertilsson S. Berglund O. Karl D. M. Chisholm S. W. (2003). Elemental composition of marine *Prochlorococcus* and *Synechococcus*: implications for the ecological stoichiometry of the sea. *Limnol. Oceanogr.* 48 1721–1731. 10.4319/lo.2003.48.5.1721

[B4] Björkman K. Duhamel S. Karl D. M. (2012). Microbial group specific uptake kinetics of inorganic phosphate and adenosine-5’-triphosphate (ATP) in the north pacific subtropical gyre. *Front. Microbiol.* 3:189. 10.3389/fmicb.2012.00189 22701449 PMC3371651

[B5] Björkman K. Thomson-Bulldis A. L. Karl D. M. (2000). Phosphorus dynamics in the North Pacific subtropical gyre. *Aquat. Microb. Ecol.* 22 185–198. 10.3354/ame022185

[B6] Björkman K. M. Duhamel S. Church M. J. Karl D. M. (2018). Spatial and temporal dynamics of inorganic phosphate and Adenosine-5’-Triphosphate in the North Pacific Ocean. *Front. Mar. Sci.* 5:235. 10.3389/fmars.2018.00235

[B7] Blake R. E. O’Neil J. R. Surkov A. V. (2005). Biogeochemical cycling of phosphorus: insights from oxygen isotope effects of phosphoenzymes. *Am. J. Sci.* 305 596–620. 10.2475/ajs.305.6-8.596 12377050

[B8] Button D. K. Robertson B. Gustafson E. Zhao X. (2004). Experimental and theoretical bases of specific affinity, a cytoarchitecture-based formulation of nutrient collection proposed to supercede the Michaelis-Menten paradigm of microbial kinetics. *Appl. Environ. Microbiol.* 70 5511–5521. 10.1128/AEM.70.9.5511-5521.2004 15345439 PMC520905

[B9] Casey J. R. Lomas M. W. Michelou V. K. Dyhrman S. T. Orchard, Ammerman J. W. (2010). Phytoplankton taxon-specific orthophosphate (Pi) and ATP utilization in the western subtropical North Atlantic. *Aquat. Microb. Ecol.* 58 31–44. 10.3354/ame01348

[B10] Chang S. J. Blake R. E. (2015). Precise calibration of equilibrium oxygen isotope fractionations between dissolved phosphate and water from 3 to 37°C. *Geochim. Cosmochim. Acta* 150 314–329. 10.1016/j.gca.2014.10.030

[B11] Colman A. S. Blake R. E. Karl D. M. Fogel M. L. Turekian K. K. (2005). Marine phosphate oxygen isotopes and organic matter remineralization in the oceans. *Proc. Natl. Acad. Sci. U.S.A.* 102 13023–13028. 10.1073/pnas.0506455102 16141319 PMC1201620

[B12] Colman A. S. Mine A. H. (2016). “Detection of phosphohydrolytic enzyme activity through the *δ*^18^O of dissolved phosphate,” *Association for the Sciences f Limnology and Oceanography Meeting* (New Orleans, LA).

[B13] Cuhel R. Waterbury J. (1984). Biochemical composition and short term nutrient incorporation patterns in a unicellular marine cyanobacterium, *Synechococcus* (WH7803). *Limnol. Oceanogr.* 29 370–374.

[B14] Diaz J. M. Ingall E. D. (2010). Fluorometric quantification of natural inorganic polyphosphate. *Environ. Sci. Technol.* 44 4665–4671. 10.1021/es100191h 20507063

[B15] Donald K. M. Scanlan D. J. Carr N. G. Mann N. H. Joint I. (1997). Comparative phosphorus nutrition of the marine cyanobacterium *Synechococcus* WH7803 and the marine diatom *Thalassiosira weissflogii*. *J. Plankt. Res.* 19 1793–1813. 10.1093/plankt/19.12.1793 32665766

[B16] Duhamel S. Dyhrman S. T. Karl D. M. (2010). Alkaline phosphatase activity and regulation in the North Pacific Subtropical Gyre. *Limnol. Oceanogr.* 55 1414–1425. 10.4319/lo.2010.55.3.1414

[B17] Fagerbakke K. M. Heldal M. Norland S. (1996). Content of carbon, nitrogen, oxygen, sulfur and phosphorus in native aquatic and cultured bacteria. *Aquat. Microb. Ecol.* 10 15–27. 10.3354/ame010015

[B18] Fang X. Liu Y. Zhao Y. Chen Y. Liu R. Qin Q. L. (2019). Transcriptomic responses of the marine cyanobacterium *Prochlorococcus* to viral lysis products. *Environ. Microbiol.* 21 2015–2028. 10.1111/1462-2920.14513 30585375

[B19] Del Giorgio P. A. Gasol J. M. (2008). Physiological structure and single-cell activity in marine bacterioplankton. *Microb. Ecol. Oceans Sec. Ed.* 1974 243–298. 10.1002/9780470281840.ch8

[B20] Gobler C. J. Hutchins D. A. Fisher N. S. Cosper E. M. Saøudo-Wilhelmy S. (1997). Release and bioavailability of C, N, P Se, and Fe following viral lysis of a marine chrysophyte. *Limnol. Oceanogr.* 42 1492–1504. 10.4319/lo.1997.42.7.1492

[B21] Grob C. Ostrowski M. Holland R. J. Heldal M. Norland S. Erichsen E. S. (2013). Elemental composition of natural populations of key microbial groups in Atlantic waters. *Environ. Microbiol.* 15 3054–3064. 10.1111/1462-2920.12145 23663455

[B22] Grob C. Ulloa O. Claustre H. Huot Y. Alarcón G. Marie D. (2007). Contribution of picoplankton to the total particulate organic carbon concentration in the eastern South Pacific. *Biogeosciences* 4 837–852. 10.5194/bg-4-837-2007

[B23] Heldal M. Scanlan D. J. Norland S. Thingstad F. Mann N. H. (2003). Elemental composition of single cells of various strains of marine *Prochlorococcus* and *Synechococcus* using X-ray microanalysis. *Limnol. Oceanogr.* 48 1732–1743. 10.4319/lo.2003.48.5.1732

[B24] Howard-Varona C. Lindback M. M. Bastien G. E. Solonenko N. Zayed A. A. Jang H. (2020). Phage-specific metabolic reprogramming of virocells. *ISME J.* 14 881–895. 10.1038/s41396-019-0580-z 31896786 PMC7082346

[B25] Karl D. M. Björkman K. M. (2015). “Chapter 5 - Dynamics of dissolved organic phosphorus,” in *Biogeochemistry of Dissolved Organic Matter*, 2nd Edn, eds Hansell D. A. Carlson C. A. (London: Academic Press), 234–334.

[B26] Krumhardt K. M. Callnan K. Roache-Johnson K. Swett T. Robinson D. Reistetter E. N. (2013). Effects of phosphorus starvation versus limitation on the marine cyanobacterium Prochlorococcus MED4 I: uptake physiology. *Environ. Microbiol.* 15 2114–2128. 10.1111/1462-2920.12079 23387819

[B27] Liang Y. Blake R. E. (2006). Oxygen isotope signature of Pi regeneration from organic compounds by phosphomonoesterases and photooxidation. *Geochim. Cosmochim. Acta* 70, 3957–3969. 10.1016/j.gca.2006.04.036

[B28] Lindell D. Padan E. Post A. F. (1998). Regulation of ntcA expression and nitrite uptake in the marine *Synechococcus* sp. strain WH 7803. *J. Bacteriol.* 180 1878–1886. 9537388 10.1128/jb.180.7.1878-1886.1998 PMC107103

[B29] Lomas M. W. Bonachela J. A. Levin S. A. Martiny A. C. (2014). Impact of ocean phytoplankton diversity on phosphate uptake. *Proc. Natl. Acad. Sci. U.S.A.* 111 17540–17545. 10.1073/pnas.1420760111 25422472 PMC4267344

[B30] Lomas M. W. Burke A. L. Lomas D. A. Bell D. W. Shen C. Dyhrman S. T. (2010). Sargasso Sea phosphorus biogeochemistry: an important role for dissolved organic phosphorus (DOP). *Biogeosciences* 7 695–710. 10.5194/bg-7-695-2010

[B31] Lomas M. W. Swain A. Shelton R. Ammerman J. W. (2004). Taxonomic variability of phosphorus stress in Sargasso Sea phytoplankton. *Limnol. Oceanogr.* 49 2303–2309. 10.4319/lo.2004.49.6.2303

[B32] Martin P. Dyhrman S. T. Lomas M. W. Poulton N. J. Van Mooy B. (2014). Accumulation and enhanced cycling of polyphosphate by Sargasso Sea plankton in response to low phosphorus. *Proc. Natl. Acad. Sci. U.S.A.* 111 8089–8094. 10.1073/pnas.1321719111 24753593 PMC4050623

[B33] Martin P. Lauro F. M. Sarkar A. Goodkin N. Prakash S. Vinayachandran P. N. (2018). Particulate polyphosphate and alkaline phosphatase activity across a latitudinal transect in the tropical Indian Ocean. *Limnol. Oceanogr.* 63 1395–1406. 10.1002/lno.10780

[B34] Martiny A. C. Coleman M. L. Chisholm S. W. (2006). Phosphate acquisition genes in *Prochlorococcus ecotypes*: evidence for genome-wide adaptation. *Proc. Natl. Acad. Sci. U.S.A.* 103 12552–12557. 10.1073/pnas.0601301103 16895994 PMC1567916

[B35] Martiny A. C. Ma L. Mouginot C. Chandler J. W. Zinser E. R. (2016). Interactions between thermal acclimation, growth rate, and phylogeny influence prochlorococcus elemental stoichiometry. *PLoS One* 11:e0168291. 10.1371/journal.pone.0168291 27936127 PMC5148161

[B36] Martiny A. C. Pham C. T. A. Primeau F. W. Vrugt J. A. Moore J. K. Levin S. A. (2013). Strong latitudinal patterns in the elemental ratios of marine plankton and organic matter. *Nat. Geosci.* 6 279–283. 10.1038/ngeo1757

[B37] Mason C. A. Hamer G. Bryers J. D. (1986). The death and lysis of microorganisms in environmental processes. *FEMS Microbiol. Lett.* 39 373–401. 10.1111/j.1574-6968.1986.tb01867.x

[B38] Mazard S. Wilson W. H. Scanlan D. J. (2012). Dissecting the physiological response to phosphorus stress in marine synechococcus isolates (cyanophyceae). *J. Phycol.* 48 94–105. 10.1111/j.1529-8817.2011.01089.x 27009654

[B39] McCarthy M. D. Hedges J. I. Benner R. (1998). Major bacterial contribution to marine dissolved organic nitrogen. *Science* 281 231–234. 10.1126/science.281.5374.231 9657711

[B40] McGillicuddy D. J. Robinson A. R. Siegel D. A. Jannasch H. W. Johnson R. Dickey T. D. (1998). Influence of mesoscale eddies on new production in the Sargasso Sea. *Nature* 394 263–266. 10.1038/28367

[B41] Middelboe M. Lyck P. G. (2002). Regeneration of dissolved organic matter by viral lysis in marine microbial communities. *Aquat. Microb. Ecol.* 27 187–194. 10.3354/ame027187

[B42] Monaghan E. J. Ruttenberg K. C. (1999). Dissolved organic phosphorus in the coastal ocean: reassessment of available methods and seasonal phosphorus profiles from the Eel River Shelf. *Limnol. Oceanogr.* 44 1702–1714. 10.4319/lo.1999.44.7.1702

[B43] Monier A. Chambouvet A. Milner D. S. Attah V. Terrado R. Lovejoy C. (2017). Host-derived viral transporter protein for nitrogen uptake in infected marine phytoplankton. *Proc. Natl. Acad. Sci. U.S.A.* 114 E7489–E7498. 10.1073/pnas.1708097114 28827361 PMC5594687

[B44] Moore C. M. Mills M. M. Arrigo K. R. Berman-Frank I. Bopp L. Boyd P. W. (2013). Processes and patterns of oceanic nutrient limitation. *Nat. Geosci.* 6 701–710. 10.1038/ngeo1765

[B45] Moore L. Ostrowski M. Scanlan D. Feren K. Sweetsir T. (2005). Ecotypic variation in phosphorus-acquisition mechanisms within marine picocyanobacteria. *Aquat. Microb. Ecol.* 39 257–269. 10.3354/ame039257

[B46] Moore L. R. Coe A. Zinser E. R. Saito M. A. Sullivan M. B. Lindell D. (2007). Culturing the marine cyanobacterium *Prochlorococcus*. *Limnol. Oceanogr. Methods* 5 353–362.

[B47] Moran M. A. Kujawinski E. B. Stubbins A. Fatland R. Aluwihare L. I. Buchan A. (2016). Deciphering ocean carbon in a changing world. *Proc. Natl. Acad. Sci. U.S.A.* 113 3143–3151. 10.1073/pnas.1514645113 26951682 PMC4812754

[B48] Murphy J. Riley J. P. (1962). A modified single solution method for the determination of phosphate in natural waters. *Anal. Chim. Acta* 27 31–36. 10.1016/S0003-2670(00)88444-5

[B49] Orchard E. D. Benitez-Nelson C. R. Pellechia P. J. Lomas M. W. Dyhrman S. T. (2010). Polyphosphate in trichodesmium from the low-phosphorus Sargasso Sea. *Limnol. Oceanogr.* 55 2161–2169. 10.4319/lo.2010.55.5.2161

[B50] Ostrowski M. Mazard S. Tetu S. G. Phillippy K. Johnson A. Palenik B. (2010). PtrA is required for coordinate regulation of gene expression during phosphate stress in a marine *Synechococcus*. *ISME J.* 4 908–921. 10.1038/ismej.2010.24 20376102

[B51] Paytan A. McLaughlin K. (2007). The oceanic phosphorus cycle. *Chem. Rev.* 107, 563–576. 10.1021/cr0503613 17256993

[B52] Puxty R. J. Millard A. D. Evans D. J. Scanlan D. J. (2016). Viruses inhibit CO2 fixation in the most abundant phototrophs on earth. *Curr. Biol.* 26 1585–1589. 10.1016/j.cub.2016.04.036 27291056

[B53] Rao N. N. Liu S. Kornberg A. (1998). Inorganic polyphosphate in *Escherichia coli*: the phosphate regulon and the stringent response. *J. Bacteriol.* 180 2186–2193. 9555903 10.1128/jb.180.8.2186-2193.1998 PMC107147

[B54] Scanlan D. J. Mann N. H. Carr N. G. (1993). The response of the picoplanktonic marine cyanobacterium *Synechococcus* species WH7803 to phosphate starvation involves a protein homologous to the periplasmic phosphate-binding protein of *Escherichia coli*. *Mol. Microbiol.* 10 181–191. 7968514 10.1111/j.1365-2958.1993.tb00914.x

[B55] Shelford E. J. Middelboe M. Møller E. F. Suttle C. A. (2012). Virus-driven nitrogen cycling enhances phytoplankton growth. *Aquat. Microb. Ecol.* 66 41–46. 10.3354/ame01553

[B56] Temperton B. Gilbert J. A. Quinn J. P. McGrath J. W. (2011). Novel analysis of oceanic surface water metagenomes suggests importance of polyphosphate metabolism in oligotrophic environments. *PLoS One* 6:e16499. 10.1371/journal.pone.0016499 21305044 PMC3030594

[B57] Thompson L. R. Zeng Q. Kelly L. Huang K. H. Singer A. U. Stubbe J. (2011). PNAS Plus: phage auxiliary metabolic genes and the redirection of cyanobacterial host carbon metabolism. *Proc. Natl. Acad. Sci. U.S.A.* 108 E757–E764. 10.1073/pnas.1102164108 21844365 PMC3182688

[B58] Torriani-Gorini A. Yagil E. Silver S. (1994). *Phosphate in Microorganisms: Cellular and Molecular Biology.* Zondervan.

[B59] Vrede K. Heldal M. Norland S. Bratbak G. (2002). Elemental composition (C, N, P) and cell volume of exponentially growing and nutrient-limited Bacterioplankton. *Appl. Environ. Microbiol.* 68, 2965–2971. 10.1128/AEM.68.6.2965 12039756 PMC123973

[B60] Wilhelm S. W. Suttle C. A. (1999). Viruses and nutrient cycles in the Sea. *BioScience* 49:781. 10.2307/1313569

[B61] Van Mooy B. S. Moutin T. Duhamel S. Rimmelin P. Van Wambeke F. (2008). Phospholipid synthesis rates in the eastern subtropical South Pacific Ocean. *Biogeosciences* 5 133–139. 10.5194/bg-5-133-2008

[B62] Van Mooy B. S. Fredricks H. F. Pedler B. E. Dyhrman S. T. Karl D. M. Koblízek M. (2009). Phytoplankton in the ocean use non-phosphorus lipids in response to phosphorus scarcity. *Nature* 458 69–72. 10.1038/nature07659 19182781

[B63] Wu J. Sunda W. Boyle E. A. Karl D. M. (2000). Phosphate depletion in the western North Atlantic Ocean. *Science* 289 759–762. 10.1126/science.289.5480.759 10926534

[B64] Zeng Q. Chisholm S. W. (2012). Marine viruses exploit their host’s two-component regulatory system in response to resource limitation. *Curr. Biol. CB* 22 124–128. 10.1016/j.cub.2011.11.055 22244998

[B65] Zimmerman A. E. Howard-Varona C. Needham D. M. John S. G. Worden A. Z. Sullivan M. B. (2020). Metabolic and biogeochemical consequences of viral infection in aquatic ecosystems. *Nat. Rev. Microbiol.* 18 21–34. 10.1038/s41579-019-0270-x 31690825

[B66] Zweifel U. L. Hagstrom A. (1995). Total counts of marine bacteria include a large fraction of non-nucleoid- containing bacteria (ghosts). *Appl. Environ. Microbiol.* 61 2180–2185. 10.1128/aem.61.6.2180-2185.1995 16535043 PMC1388461

