# Supplementary figures and images for: Phosphorus Release and Regeneration Following Laboratory Lysis of Bacterial Cells
Source: Front Microbiol. 2021 Apr 8;12:641700. doi: 10.3389/fmicb.2021.641700 (PMC8060472; doi:10.3389/fmicb.2021.641700)

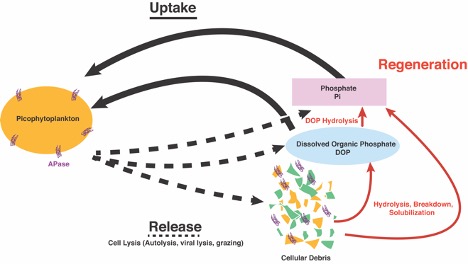

Supplement: Supplementary Figure 1 — Conceptual Diagram of Cellular Phosphorus Cycling: Our study focuses on quantifying and mechanistically understanding phosphorus release and regeneration fluxes within the microbial loop. The major fluxes at the cellular level for phosphorus cycling are shown above alongside the pathways and reactions that transform phosphorus between phosphorus pools. [file Image_4.JPEG]

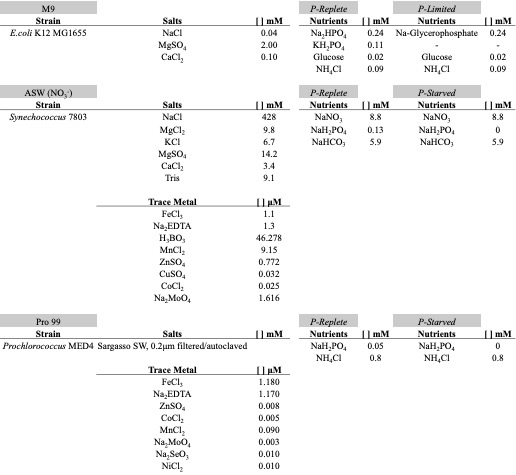

Supplement: Supplementary Table 1 — Media Composition (Lindell et al., 1998; Moore et al., 2007). [file Image_1.JPEG]

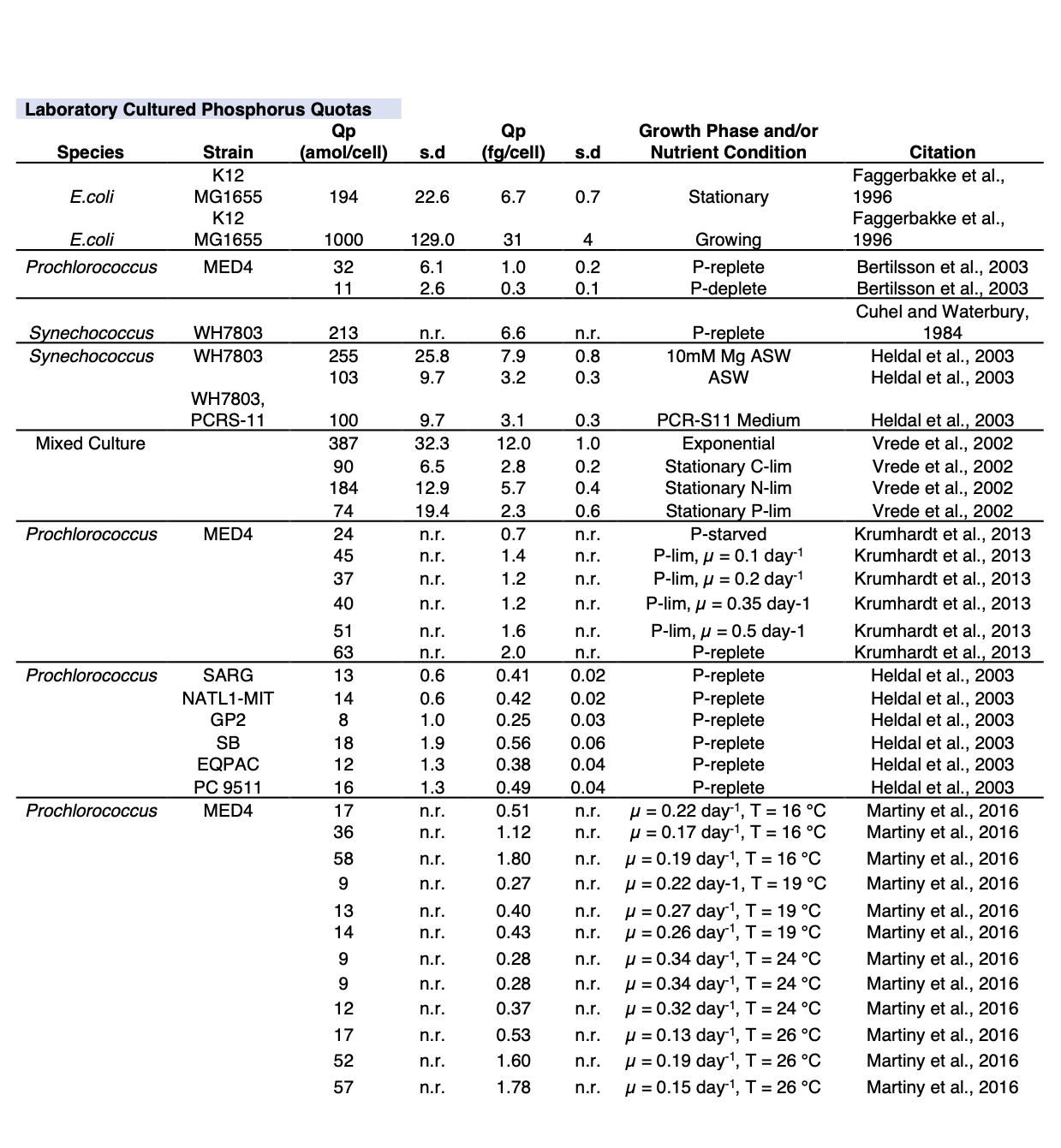

Supplement: Supplementary Table 2 — Compilation of Cultured Cellular P Quotas: Collection of published cellular p contents (Qp) for Prochlorococcus and Synechococcus strain [file Image_2.TIFF]

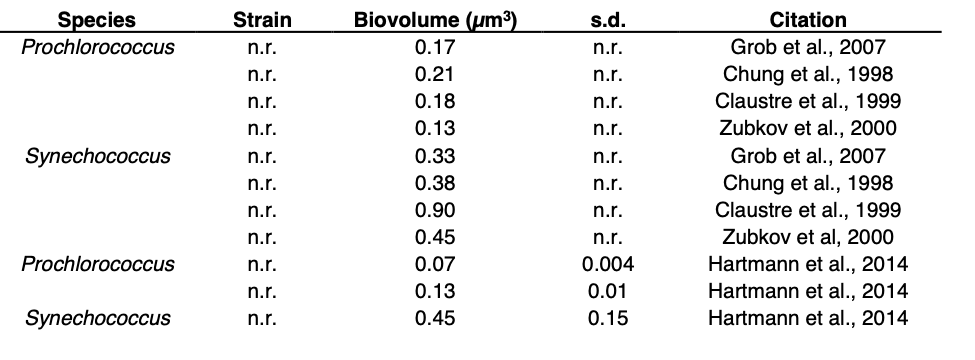

Supplement: Supplementary Table 3 — Compilation of Cultured Cyanobacterial Biovolumes: Collection of published cellular biovolume for surface ocean populations of Prochlorococcus and Synechococcus. Not reported – (n.r). [file Image_3.TIFF]
