# Supplementary material for: Phosphorus Release and Regeneration Following Laboratory Lysis of Bacterial Cells
Source: Front Microbiol. 2021 Apr 8;12:641700. doi: 10.3389/fmicb.2021.641700 (PMC8060472; doi:10.3389/fmicb.2021.641700)
Supplement: Supplementary Table 4 — Summary of Experiments: Below is a tabular presentation of the experiments performed, alongside the analyses and treatments for each strain studied. [file Data_Sheet_1.PDF]

Strain	Nutrient Condition	DOP Spike	Phosphorus Assay	
			TDP	SRP
E.coli K-12 MG1655	P-Replete	5'AMP, GYP, RNA, Pyrophosphate		X
	P-Deplete	5'AMP, GYP, RNA		X
Synechococcus sp. WH7803	P-Replete	GYP	X	X
	P-Deplete	GYP	X	X
Prochlorococcus sp. MED4	P-Replete	GYP	X	X
	P-Deplete	GYP	X	X

APase Assay
X
X
X
X
X
X
